# Laying the foundations for hepatitis C elimination: evaluating the development and contribution of community care pathways to diagnostic efforts

**DOI:** 10.1186/s12889-022-14911-1

**Published:** 2023-01-07

**Authors:** Emma Robinson, Christopher J. Byrne, James Carberry, Andrew Radley, Lewis J. Beer, Sarah K. Inglis, Jan Tait, Iain Macpherson, David Goldberg, Sharon J. Hutchinson, Matthew Hickman, John F. Dillon

**Affiliations:** 1 Department of Gastroenterology, NHS Tayside, Ninewells Hospital and Medical School, Dundee, UK; 2 Division of Molecular and Clinical Medicine, School of Medicine, University of Dundee, Ninewells Hospital and Medical School, Dundee, UK; 3 Directorate of Public Health, Kings Cross Hospital, NHS Tayside, Dundee, UK; 4 Tayside Clinical Trials Unit, School of Medicine, University of Dundee, Ninewells Hospital and Medical School, Dundee, UK; 5 Public Health Scotland, Meridian Court, Glasgow, UK; 6 School of Health and Life Sciences, Glasgow Caledonian University, Glasgow, UK; 7 Population Health Sciences, Bristol Medical School, University of Bristol, Bristol, UK

**Keywords:** Hepatitis C virus, Models of care, People who inject drugs

## Abstract

**Background:**

Hepatitis C Virus (HCV) is a public health threat which contributes substantially to the global burden of liver disease. There is much debate about effective approaches to scaling up diagnosis of HCV among risk groups. Tayside, a region in the East of Scotland, developed low-threshold community pathways for HCV to lay the foundations of an elimination strategy. In this retrospective study, we sought to: quantify the contribution of community pathways to increasing HCV diagnosis; understand if shifting diagnosis to community settings led to a higher proportion of individuals tested for HCV being actively infected; and describe functional characteristics of the care pathways.

**Methods:**

Descriptive statistics were used to for analysis of routinely-collected HCV testing data from 1999 to 2017, and a review of the development of the care pathways was undertaken. Community-based testing was offered through general practices (GP); nurse outreach clinics; prisons; drug treatment services; needle and syringe provision (NSP) sites; community pharmacies; and mosques.

**Results:**

Anti-HCV screening was undertaken on 109,430 samples, of which 5176 (4.7%) were reactive. Of all samples, 77,885 (71.2%) were taken in secondary care; 25,044 (22.9%) in GPs; 2970 (2.7%) in prisons; 2415 (2.2%) in drug services; 753 (0.7%) in NSPs; 193 (0.2%) pharmacies; and 170 (0.1%) in mosques. The highest prevalence of HCV infection among those tested was in NSP sites (26%), prisons (14%), and drug treatment centres (12%).

**Conclusions:**

Decentralised care pathways, particularly in harm reduction and other drug service settings, were key to increasing diagnosis of HCV in the region, but primary and secondary care remain central to elimination efforts.

## Background

Hepatitis C Virus (HCV) is a public health threat which contributes substantially to the global burden of liver disease. Approximately 58 (95%CI 46–76) million people are estimated to be chronically infected with HCV, with as many as 1.5 (1.1–2.6) million incident infections each year, and 290,000 (95%CI 230–580) people dying annually consequent to HCV-related causes [1]. Following the availability of safe and highly effective Direct Acting Antiviral (DAA) treatment for HCV, elimination of the infection as a public health threat became a tangible goal and the World Health Organization, in 2016, released an implementation strategy designed to facilitate this, underpinned by an approach of improving access to HCV diagnosis and treatment for key populations using novel strategies [2]. More recently published WHO guidelines have explicitly supported task-shifting of HCV diagnosis and management into decentralised, community environments, to further enfranchise infected individuals into the HCV care cascade [3].

Scotland rapidly adopted the WHO elimination aims, seeking to achieve it – defined as no greater than 1 in 1000 people chronically infected with HCV – by 2024, six years prior to the WHO target [4]. Concurrent to the rapid adoption of this new approach, underpinned by a community-embedded care ethos, and access to safe, effective, DAAs, National Health Service (NHS) Tayside, a large health board in the East of Scotland, initiated a natural experiment of diagnosis and treatment of HCV with the aim of facilitating elimination of HCV as a public health threat in the region [5]. Initial results from the treatment phase of this work suggested the programme had achieved its aims of diagnosing and treating most chronically infected People Who Inject Drugs (PWID) in the area, who represented the primary infected population [6]. Prior to this treatment phase (2017–20), multiple decentralised diagnostic care pathways were iteratively developed to improve outreach to potentially infected individuals in the region.

Given the drive for elimination has generated much debate about how to deliver on the WHO aim to diagnose a least 90% of HCV-infected people, particularly in contexts where prevalence is not high enough to justify population-level screening, and the substantial gaps that remain in closing the diagnosis gap in HCV [7], we sought to quantify the relative contributions of multiple targeted community pathways to HCV diagnostic access in Tayside over time. The aim of this analysis is to provide a useful description of an effective combination of care pathways to diagnose HCV infection in a typical high-income Western population, and therefore aid others aiming to take a similar public health approach. Specifically, this study aims to: quantify the relative contribution of community care pathways to increasing HCV diagnosis in Tayside from 1999 to 2017; understand if shifting diagnosis from tertiary to community settings led to a higher proportion of individuals tested for HCV being actively infected; and describe both the evolution, and the functional aspects, of the pathways and environments they were embedded in over the study period.

## Methods

### Study setup

This study is described on clinicaltrials.gov (NCT03513796, registered 02/05/2018). Ethical approval was received from West of Scotland Research Ethics Committee (18/WS/0035). The study was co-sponsored by University of Dundee and Tayside Health Board (2016CO01). Caldicott Guardian approval – a procedure that ensures the protection and appropriate use of patient-identifiable data – was received for data access (IGTCAL4762), which was retrieved from NHS Tayside HCV clinical databases and NHS Tayside Virology department.

### Measures

This was a retrospective analysis using routinely collected clinical data. Individuals were differentiated using their Community Health Index (CHI) number, a unique identifier allocated to every registered NHS patient in Scotland, where available. De-identified data were stored using Microsoft Excel 2013 and held on secure servers with controlled access. The following data were collected: CHI (where available); testing source and testing year; and results of anti-HCV and HCV RNA testing.

### Study setting

Tayside is a defined geographic region in the East of Scotland with a population of approximately 416,000 people. Residents’ health needs are served by NHS Scotland, which is free-of-charge at the point of delivery. The region is demographically and socio-economically diverse; home to some of the wealthiest, and most deprived, areas of Scotland. Since 2004, blood-borne virus (BBV) services have been led regionally through a Managed Care Network (MCN), which brings together NHS services, the charity sector, higher education, and partners from governmental and third-sector agencies, to enable a multi-dimensional approach to service delivery [8]. Previous analyses have demonstrated this approach – paired with the introduction of Dried Blood Spot (DBS) sampling for HCV – increased access to HCV care and reduced all-cause mortality in the HCV-infected population in Tayside [9]. The region had a historically high burden of chronic HCV, consequent to the prevalence of IDU and absence of primary prevention measures [10].

### Care pathways

**Fig. 1:**
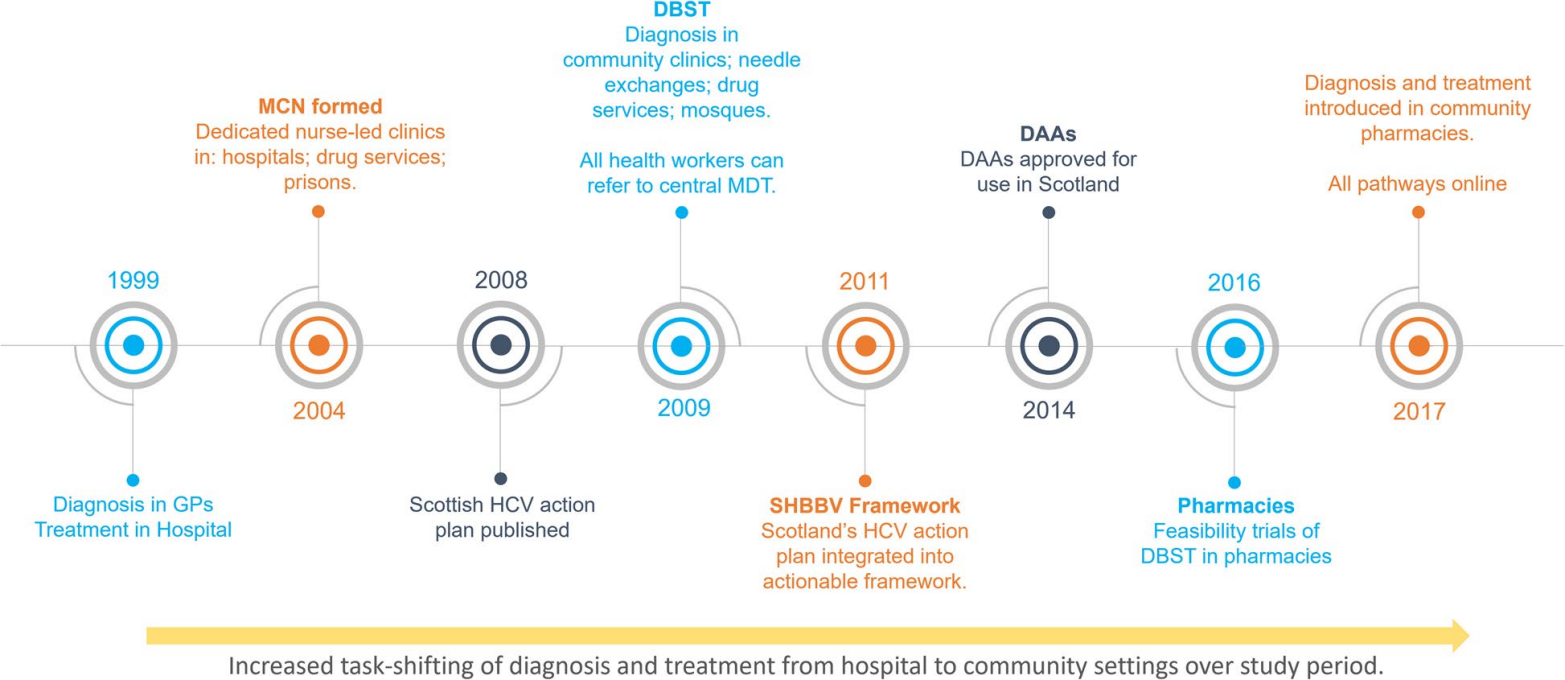
Chronological development of community pathways in Tayside, 1999–2017. Abbreviations: GP, general practitioner; MCN, managed care network; DBST, dried blood spot testing; MDT, multi-disciplinary team; SHBBVF, Sexual Health & Blood-borne Virus Framework; DAA, direct acting antivirals

As mentioned, the viral hepatitis service, and the care pathways its activities are administered through, were iteratively developed, concurrent to evolving local research, and wider diagnostic, and therapeutic advances (Fig. 1), primarily DBS sampling and access to DAAs, which enfranchised a wider pool of testers and prescribers [8, 9, 11–16].

Over the course of the study period, given the large sub-population of Pakistani descent in Dundee, among whom there is likely to be a proportionately higher prevalence of HCV, a time-limited outreach pathway was also delivered through local mosques (Fig. 1) which included several awareness raising events and testing and prescribing pop-up clinics [17, 18].

**Fig. 2:**
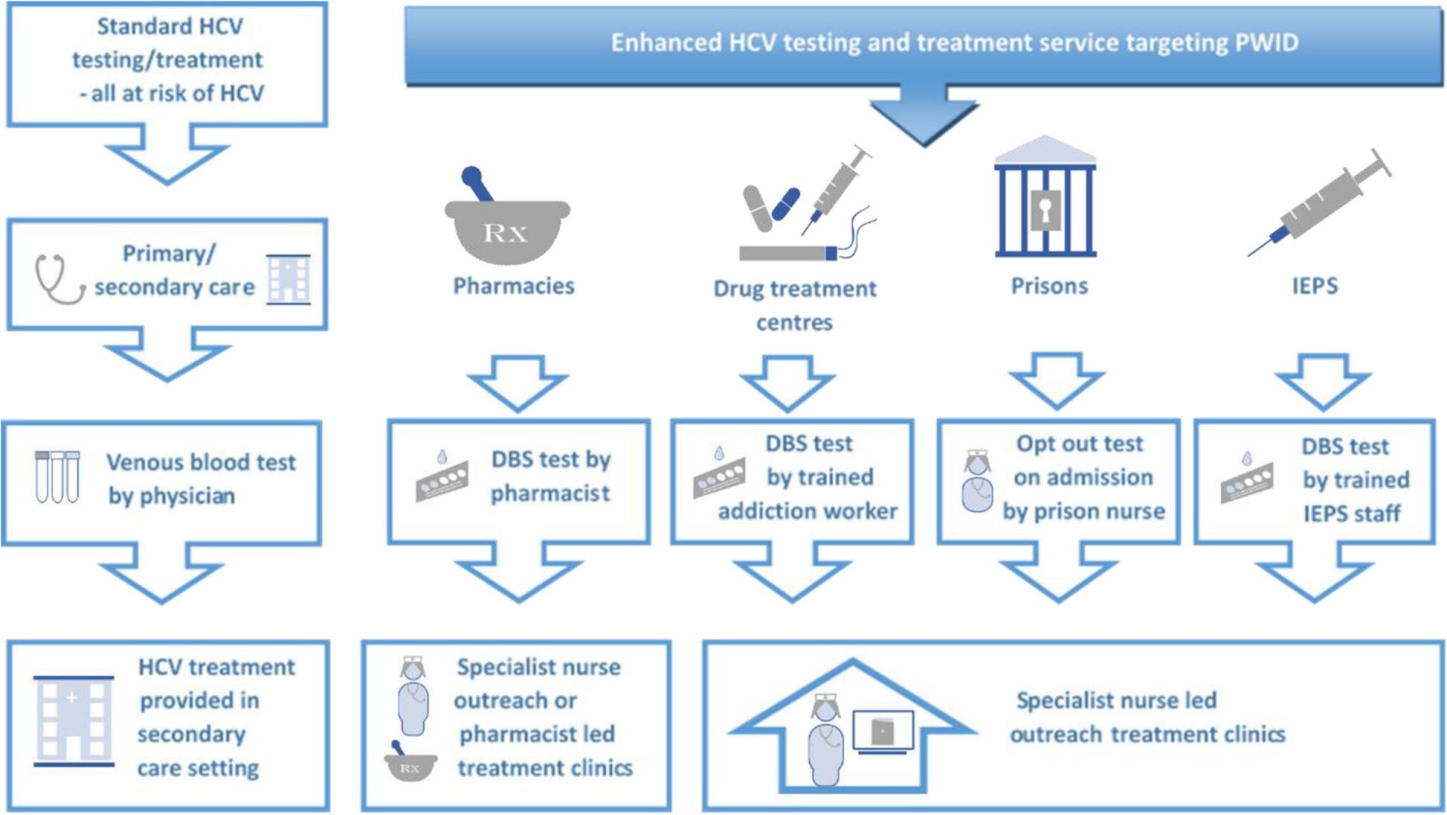
Functional aspects of care pathways for hepatitis c virus, NHS Tayside, 2017. Abbreviations: HCV, hepatitis c virus; PWID, people who inject drugs; IEPS; injection equipment provision sites, otherwise known as needle and syringe provision (NSP) sites; DBS, dried blood spot

At the end of the study period, immediately prior to the treatment scaleup phase among PWID in Tayside (reported elsewhere) [6], HCV testing and treatment with DAAs was available through primary care/secondary care; drug treatment services; needle and syringe provision (NSP) sites, community pharmacies; and the prison estate, aligned with national guidelines [19]. The functional features of these pathways are outlined in Fig. 2. In the primary/secondary care interface pathway, conventional phlebotomy is undertaken by general practice staff, who target those with identified risk factors for infection (e.g. known IDU; historic blood transfusion; migration from a high prevalence country; infection with other BBVs; needle-stick injuries; unsafe tattooing), with onward referral to secondary care [19]. Additionally, since 2015, individuals with elevated liver function tests identified through GP testing, undergo automatic HCV screening using the same sample [20]. In community pharmacies, pharmacists test Opioid Agonist Therapy (OAT) clients for HCV using DBS sampling and can prescribe DAAs either as an independent prescriber or through a Patient Group Direction (PGD). In drug treatment centres, clients are offered routine testing by conventional or DBS sampling by support workers and nursing staff; those who are independent prescribers can prescribe DAAs, others can refer into a central Multi-Disciplinary Team (MDT) for further management. In prisons, opt-out testing for HCV is in place using conventional and DBS methods, with independent prescribing available on site. Complex cases can be referred to MDT for discussion. Similarly, in NSPs, support workers and nursing staff offer testing with on-site prescribing with MDT support.

### Statistics

Descriptive statistics to derive counts and proportions were undertaken using IBM Statistical Product and Service Solutions (SPSS) 22. Figures were created with Microsoft Excel 2013.

## Results

From 1999 to 2017 inclusive, 109,430 samples of any type were screened for HCV antibodies, of which 5176 (4.7%) were reactive. A total of 16,205 samples were screened for HCV RNA, of which 7332 (45%) had HCV RNA above the limit of detection (10 IU/mL). Note, these figures include repeat samples. Of all anti-HCV samples processed, 77,885 (71.2%) were administered across 432 secondary care environments (a diverse range of wards and specialities); 25,044 (22.9%) were undertaken in 120 GP practices (many no longer operating); 2970 (2.7%) were done within three prisons; 2415 (2.2%) within three drug treatment services; 753 (0.7%) within NSP sites; 193 (0.2%) in 25 community pharmacies; and 170 (0.1%) within three mosques.

**Fig. 3:**
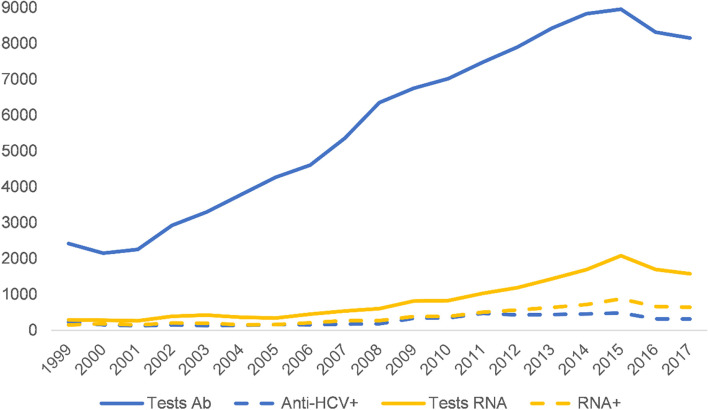
Annual trends in hepatitis c virus testing and test reactivity from 1999 to 2019, NHS Tayside. Abbreviations: GP, general practitioner; NSPs, needle and syringe provision sites; AB, antibody; HCV, hepatitis c virus; RNA, ribonucleic acid

**Fig. 4:**
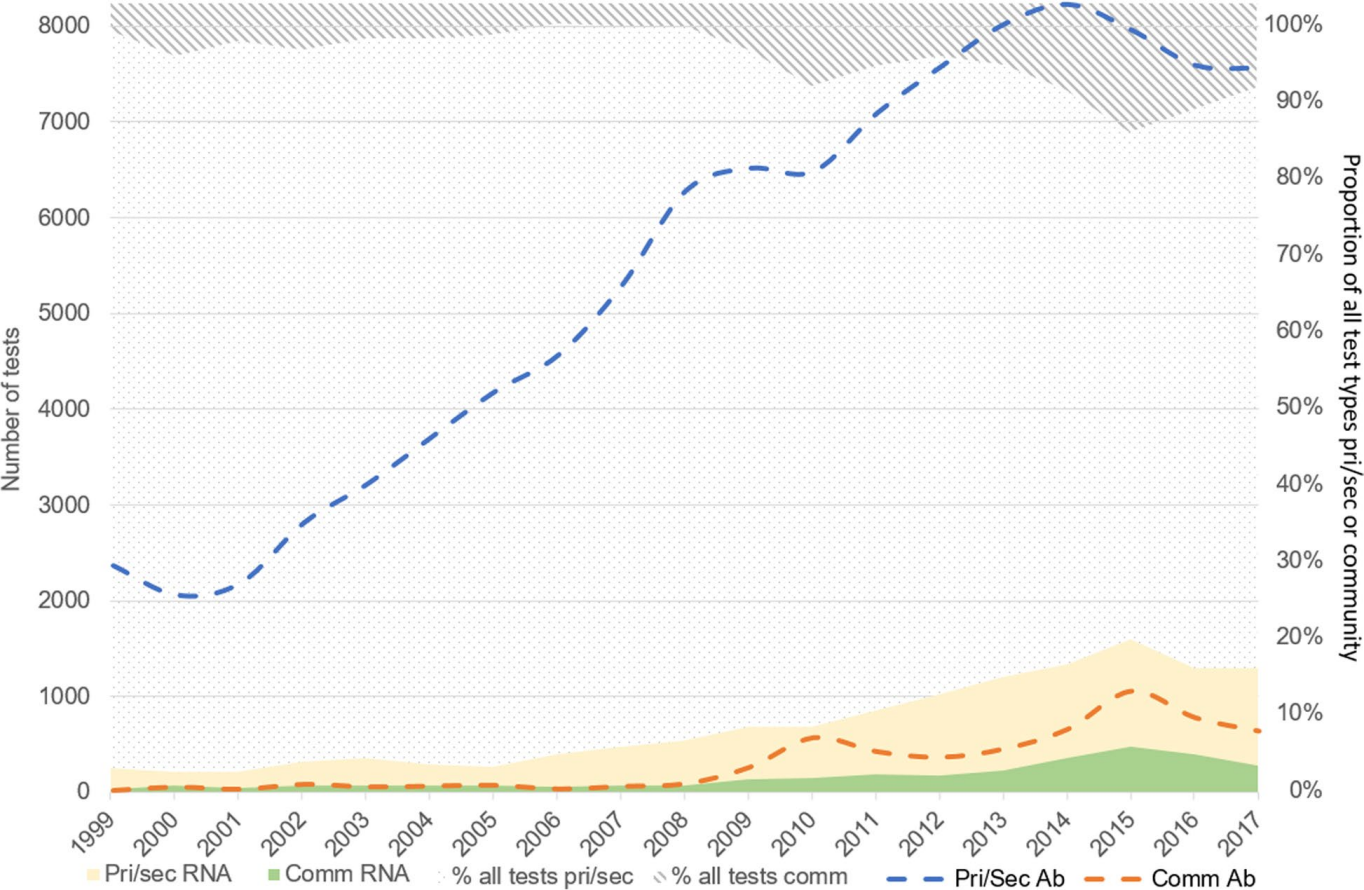
Trends in HCV antibody and RNA testing demonstrating increasing contribution of newly-established community pathways of care in management of HCV diagnosis and treatment, Tayside, 1999–2017. Abbreviations: Pri/Sec, primary and secondary care pathways; Comm, community pathways; RNA, ribonucleic acid; Ab, antibody. Note: Greyscale shaded area shows proportion of all tests (Ab and RNA) in conventional or community setting

Anti-HCV and HCV RNA detection varied over time, with the absolute quantity of tests undertaken increasing following the introduction of the MCN structure in 2004 with related nurse- and pharmacist-led community approaches over time (Fig. 3). When assessing the contribution of HCV diagnosis within community pathways relative to conventional care environments, a temporal increase in the proportion of testing was observed. This was more pronounced for RNA testing, compared to anti-HCV screening (Fig. 4) – alongside an increasing proportion of all tests occurring in community environments – which reflects the growing monitoring and management of HCV treatment in community settings over time.

**Fig. 5:**
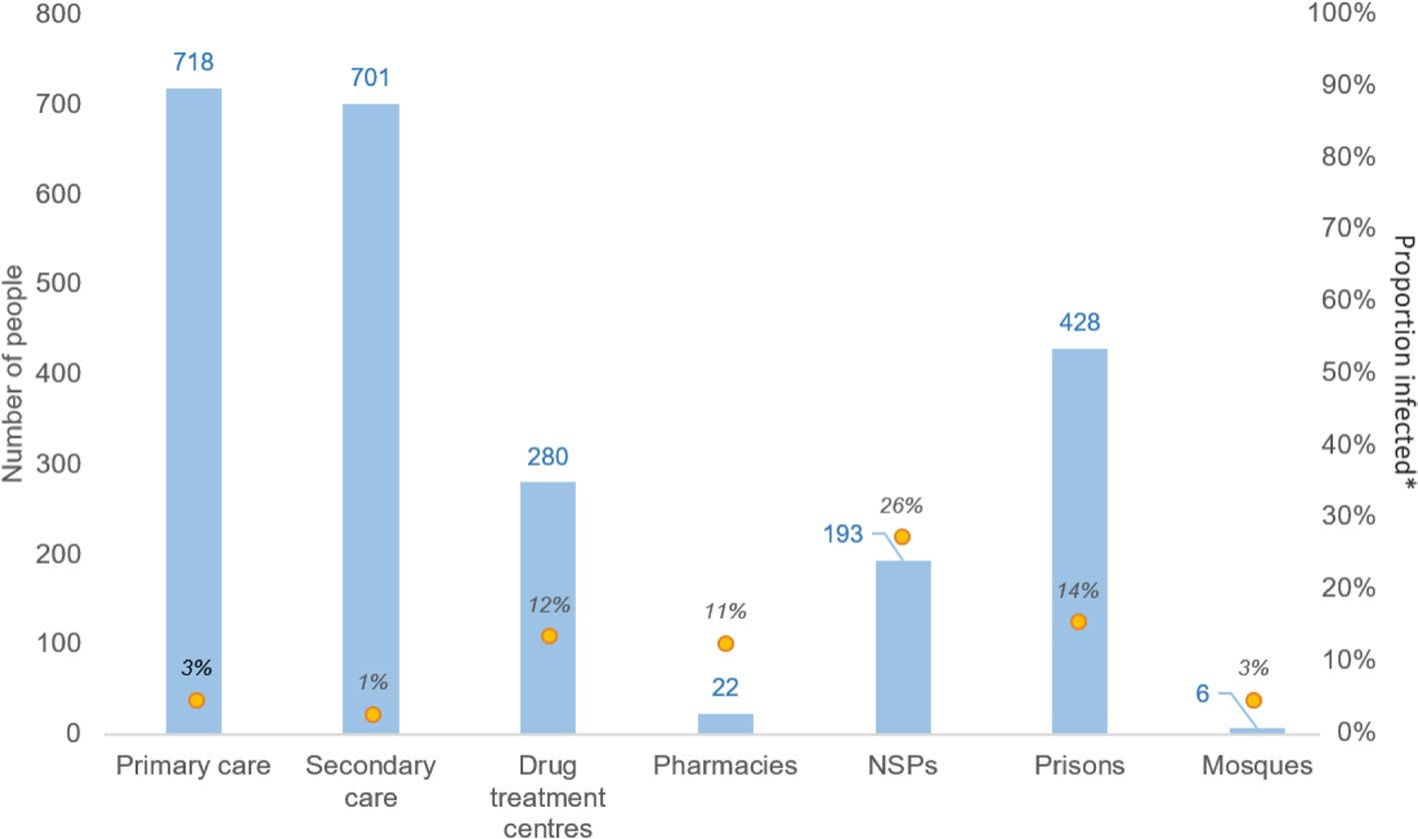
Number of individuals with detectable HCV RNA across each pathway from 1999 to 2017 in Tayside with estimates of proportionate levels of HCV RNA positivity relative to total number of anti-HCV tests administered in each pathway. *Actively infected individuals as a proportion of all anti-HCV tests administered. Notes: Blue bars are number of actively infected individuals diagnosed in each pathway over the study period. Yellow dots are proportion of actively infected individuals relative to number of anti-HCV screening tests undertaken in that pathway in the study period. Numerous/denominators are as follows: primary care (*n* = 718/24,969); secondary care (*n* = 701/77,885); drug treatment services (*n* = 280/2415); pharmacies (*n* = 22/193); NSPs (*n* = 193/753); prisons (*n* = 428/2970); mosques (*n* = 6/177). Important: total diagnosed sums to 2348. This is all individuals including those who may have migrated from Tayside, been transiting through local prisons, or become otherwise ineligible for onward treatment locally. Abbreviations: NSPs, needle and syringe provision sites

To understand whether each pathway was reaching the population intended, HCV RNA detection, as a proportion of anti-HCV tests undertaken, was calculated. The highest raw quantity of individuals were diagnosed in primary and secondary care, followed by prisons, drug treatment services, NSPs, pharmacies, and lastly the time-limited mosque outreach pathway (Fig. 5). However, the highest proportionate prevalence of active infections were all observed in community pathways, and found to be particularly pronounced in NSPs, prisons and drug treatment centres.

The total number of HCV diagnoses of any aetiology – including those who may have migrated from Tayside, been transiting through local prisons, or subsequently died – over the study period sums to 2348; of those, 1419 (60.4%) diagnoses occurred in conventional primary or secondary care settings, whilst 929 (39.6%) occurred in decentralised community-embedded pathways.

## Discussion

This retrospective study of routinely collected HCV testing data spanning a 19-year period has illustrated a substantial increase in volume, access to testing for, and attendant diagnosis of, HCV concurrent to multiple service developments over time. Testing activity peaked around the 2014–16 period, immediately prior to Tayside’s HCV treatment scale-up phase among PWID. Screening within the conventional healthcare pathways – primary and secondary care – showed a large volume of testing without a corresponding large proportion of cases diagnosed which suggests these pathways were not effectively reaching the primarily infected population which, in Tayside, is known to be PWID [10]. Through developing community pathways embedded within environments frequently accessed by PWID, Tayside has been able to bring targeted low-threshold diagnosis to most HCV-infected people in the region, thereby effectively laying the foundations for work which was recently shown to have led to the substantive elimination of HCV as a public health threat in the area [6, 21].

The community diagnostic pathways, as described (Fig. 2), represent a long-term coordinated regional strategy with an emphasis on bringing HCV care to where people are, to close the gaps in access to testing and onward care for those most at risk. Pre-dating the formal endorsement of a task-shifting approach to HCV by the WHO [1, 2], many of the pathways described here began life as collaborative research studies. In NSPs, diagnosis and treatment with Pegylated-interferon (Peg-IFN) was offered to service clients, which showed that it was feasible to deliver even arduous older therapies to actively-injecting PWID, many of whom reported no fixed abode, with high treatment completion rates [11]. This led to a subsequent DAA-based trial which was similarly successful and the adoption of the pathway as standard care within the wider Tayside portfolio [22, 23]. Within pharmacies, initial research indicated opioid agonist therapy (OAT) clients preferred to access HCV care in their community pharmacy, which led to feasibility studies of DBS testing in this setting – an effective approach – and subsequent trials which demonstrated pharmacist-led HCV testing and treatment resulted in OAT clients being twice as likely to agree to a test, 88% more likely to initiate treatment, and twice as likely to achieve a cure [12, 13, 15, 16, 24]. This pathway was later developed further by offering point-of-care RNA testing, which was also shown to be effective, and pharmacies now form a core stream within the pathway portfolio in Tayside [25, 26]. Drug treatment and prison pathways were collaboratively developed through partnerships formed in the MCN, which involved training both non-specialist healthcare, and non-healthcare, staff to test and refer for HCV using DBS sampling, and concurrent to the evidence generating processes undertaken within other pathways.

It is noteworthy that the absolute quantity of HCV testing undertaken increased with the addition of each new pathway (Fig. 3). We believe that widening the pool of diagnostic avenues ameliorated previously identified disparities in geographic access to care in Tayside [27]. Interestingly, as the community pathways became functional over time, the rates of HCV screening and diagnosis in the primary/secondary care pathway did not attenuate substantially. The proportion of all testing attributed to community pathways, which grew over time (Fig. 4), more substantially for RNA testing, suggests that the primary/secondary care axis and the community pathways were reaching diverging populations over the study period. The high RNA positivity relative to all anti-HCV testing (Fig. 5) in community pathways compared to conventional care supports this inference, with proportionally more individuals testing positive in pathways accessed by PWID. This aligns with approaches taken elsewhere, for example in Georgia where a national elimination scheme implemented via NSPs, prisons, and homeless shelters, has improved diagnosis and treatment of HCV among PWID, the primary infected population [28], and in Iceland where devolved HCV care pathways, for example through prisons and homeless shelters in addition to conventional care, have led to the effective elimination of HCV as a public health threat in an epidemic characterised, like Tayside’s, by IDU [29].

Recent work has demonstrated a close relationship between decreasing prevalence and reducing incidence of HCV, with prevalence measurements being a more practical approach to take [30]. Assuming a historic Scottish chronic HCV population prevalence of approximately 0.5–0.6% is reflective of the epidemic in Tayside [4] – albeit the primary risk factor for infection locally is IDU, meaning the actual prevalence may not track this older national estimate – around 2000–2500 individuals could have been infected with HCV locally over the study period. Based on this estimate, notwithstanding its limitations, the total number of diagnoses made over the study period is suggestive that Tayside has made substantial inroads to diagnosing HCV at the population level through the regional matrix of diagnostic pathways. In light of recent figures which demonstrate most countries are not progressing well towards elimination by 2030 due to bottlenecks in linkage to diagnosis and onward care [31], we would suggest that the multi-agency, integrated approach which shifted the majority of diagnostic activities out of hospital settings and into community settings can be an effective strategy. This would align well with any planned regional micro-elimination approach, recently demonstrated to be effective in linking critical risks groups to HCV cure [32, 33].

This study has multiple limitations. First, the use of routine administrative healthcare data is open to biases and potential errors, including mischaracterisation at input and linkage problems [34]. We tried to ameliorate these where possible by using CHI numbers, minimising the data collected at the patient level, and manually investigating discrepancies where possible. Given the sample size, however, we cannot offer complete certainty regarding potential errors or biases relating to the quality of the data, including possible duplicate entries due to missing CHI numbers. Further, it was not possible to link all tests to individuals, so in the primary analysis the results reported likely include re-sampling of the same individual(s) (e.g., on-treatment response check, end of treatment). The likelihood of re-sampling was lower for certain pathways (primary/secondary care, mosque) relative to others where it was higher (prisons, NSPs, drug treatment centres, pharmacies), due to differences in risk and the way treatment delivery changed over time. The proportion of all tests conducted in pathways with lower likelihood of resampling (94.2%) relative to those with higher likelihood (5.8%) suggests the overall proportion of duplicates is likely to be low. Coupling this likelihood with the main analysis focussing on assessing the introduction of testing in novel environments, rather than on individual outcomes, we do not feel the risk of repeat testing negatively impacts the overall message of the manuscript. Beyond this, some individuals could have been diagnosed prior to 1999, but been counted as diagnoses in subsequent years. In applying the national prevalence estimate to Tayside in the Discussion, we acknowledge this is a crude approach; work with linked administrative datasets to quantify the actual prevalence and incidence of HCV in Tayside is forthcoming to address this limitation. Therefore, we have been purposefully cautious in interpreting this. Finally, we have not undertaken a cost-efficacy analysis with respect to offering testing within each of these pathways. This work is the focus of dedicated ongoing research which will be reported as part of a wider analysis using comparator regions [5].

## Conclusions

From 1999 to 2017, NHS Tayside iteratively developed and implemented novel decentralised HCV care pathways in low-threshold environments which improved access to HCV diagnosis for the primary affected population in the region. This strategy appears to have been effective in helping services to reach HCV-infected individuals who had previously not been engaged by conventional health services. Other districts in high-income Western settings might consider a similar approach as part of an elimination strategy to improve diagnostic outreach, where the HCV epidemic is characterised by risk related to IDU.

## Data Availability

The data supporting this study were obtained from routinely updated NHS health records in line with approval granted by the NHS Caldicott Guardian. The individuals to whom the data pertains did not explicitly consent to its use for research purposes. Therefore, it is not possible for the authors to share this data. Nevertheless, interested parties may submit requests for relevant data to NHS Tayside Information Governance by email on: informationgovernance.tayside@nhs.scot.

## Abbreviations

BBV: Blood-borne Virus
CHI: Community Health Index
DAA: Direct Acting Antiviral
DBS: Dried Blood Spot
HCV: Hepatitis C Virus
IDU: Injection Drug Use
IU/mL: International Units per millilitre
MCN: Managed Care Network
MDT: Multidisciplinary Team
NHS: National Health Service
NSP: Needle and Syringe Provision
OAT: Opioid Agonist Therapy
Peg-IFN: Pegylated Interferon
PGD: Patient Group Direction
PWID: People Who Inject Drugs
SPSS: Statistical Product and Service Solutions
